# Outcomes in children with rheumatic diseases following COVID-19 vaccination and infection: data from a large two-center cohort study in Thailand

**DOI:** 10.3389/fped.2023.1194821

**Published:** 2023-06-08

**Authors:** Butsabong Lerkvaleekul, Sirirat Charuvanij, Maynart Sukharomana, Kwanchai Pirojsakul, Malisa Kamolwatwong, Soamarat Vilaiyuk

**Affiliations:** ^1^ Division of Rheumatology, Department of Pediatrics, Faculty of Medicine Ramathibodi Hospital, Mahidol University, Bangkok, Thailand; ^2^ Division of Rheumatology, Department of Pediatrics, Faculty of Medicine Siriraj Hospital, Mahidol University, Bangkok, Thailand; ^3^ Division of Nephrology, Department of Pediatrics, Faculty of Medicine Ramathibodi Hospital, Mahidol University, Bangkok, Thailand

**Keywords:** disease flare, SAR-CoV-2, autoimmune diseases, immunization, new-onset of rheumatic disease, juvenile idiopathic arthritis, prednisolone, connective tissue diseases

## Abstract

**Introduction:**

Vaccination against coronavirus disease 2019 (COVID-19) is effective in protecting patients from severe COVID-19 infection. Disease flare-up following immunization in children with rheumatic disorders may result in patient reluctance to receive the vaccine. Underlying rheumatic diseases or the use of immunosuppressive drugs may influence the outcomes of COVID-19 vaccination and infection. We aimed to describe outcomes in children with rheumatic diseases following COVID-19 immunization and infection.

**Methods:**

This retrospective study was performed at two large academic centers in Thailand. During the COVID-19 pandemic, all patients were routinely queried about COVID-19-related conditions. We included patients with rheumatic diseases aged <18 years who received at least one dose of a COVID-19 vaccine or had a history of COVID-19 infection with more than 6 months of recorded follow-up after the last vaccine dose or COVID-19 illness. Demographic information and data on clinical symptoms, disease activity, treatment, outcomes, and COVID-19 vaccination and infection were collected.

**Results:**

A total of 479 patients were included. Most (229; 47.81%) patients had juvenile idiopathic arthritis, followed by connective tissue diseases (189; 39.46%), vasculitis syndromes (42; 8.76%), and other rheumatic diseases (19; 3.97%). Approximately 90% of patients received at least one dose of COVID-19 vaccination, and half of the patients had COVID-19 infection. Among patients, 10.72% and 3.27% developed a flare after COVID-19 vaccination and COVID-19 illness, respectively. Flare severity after COVID immunization and infection was mainly mild to moderate. The predictor of flare after COVID-19 vaccination was the use of prednisolone ≥10 mg/day before vaccination (hazard ratio: 2.04, 95% confidence interval: 1.05–3.97, *p* = 0.037). Inactive disease before receiving the COVID-19 vaccination was a predictor of inactive status after a flare (hazard ratio: 2.95, 95% confidence interval: 1.04–8.40; *p* = 0.043). Overall, 3.36% and 1.61% of patients experienced a new onset of rheumatic disease after receiving the COVID-19 vaccine and after COVID-19 infection, respectively.

**Conclusion:**

The COVID-19 vaccine is recommended for children with rheumatic disease, particularly those who are in stable condition. After COVID-19 vaccination, patients—especially those with active disease before vaccination or those receiving concurrent prednisolone doses of ≥10 mg/day—should be closely monitored.

## 1. Introduction

During the global coronavirus disease 2019 (COVID-19) pandemic, over 600 million cases of COVID-19 infection were reported globally and over 6.7 million people died—based on data until February 2023 (1). Approximately 4.7 million Thais, including 774,591 children (379,686 children aged <10 years and 394,905 children aged between 10 and 19 years) contracted COVID-19 (2). Of 33,902 fatalities, 126 were children less than 10 years of age and 93 were children 10–19 years of age, indicating that the mortality rate among children was lower than that of adults (1, 3). Although the Thai government implemented several measures to prevent the COVID-19 outbreak (4), vaccination has been the core strategy to prevent COVID-19 infection or lessen its severity. In February 2021, the inactivated vaccine CoronaVac (Sinovac Biotech, Beijing, China) was the first COVID-19 vaccine to launch in Thailand, followed by the inactivated vaccine CorrBBIBP-CorV (Sinopharm, Shanghai, China) and the adenovirus-based vaccine ChAdOx1 nCoV-19 (AstraZeneca, Oxford, UK). In September 2021, the Thai government officially implemented a vaccination campaign with the mRNA vaccine BNT162b2 (Pfizer-BioNTech, New York, NY, USA) for children 12 years and older (5), followed by children aged 5 to 11 years in January 2022 (6), and children aged between 6 months and less than 5 years in October 2022 (7). The mRNA vaccine mRNA1273 (Moderna, Cambridge, MA, USA) is the most recently introduced mRNA vaccine in Thailand and is recommended for children 6 years and older (8).

Most Thai children received BNT162b2, with some parents preferring that their children receive only inactivated vaccines such as CoronaVac or BBIBP-CorV. According to The Royal College of Pediatrics of Thailand, the recommended regimen of BNT162b2 for immunocompromised children is an initial three doses and one booster dose for children aged 5–11 years and two booster doses for children aged 12 to less than 18 years (8). Some children are allowed combination regimens of inactivated and mRNA vaccines. We discovered that approximately 80% of children aged 12–17 years and roughly 50% of children aged 5–11 years received a second dose of the COVID-19 vaccine after the COVID-19 vaccination campaign for children was launched. Only 2% of children aged between 6 months to less than 5 years received the second vaccine dose (9). Following the first detection of the Omicron variant in South Africa in November 2021, approximately 90% of all COVID-19 strains in Thailand were Omicron by February 2022, growing to 100% in April 2022 (10). Although the Omicron variant is less virulent and rarely causes severe infection, it is highly transmissible—especially in school-aged children (11). Because schools reopened in May 2022, the number of infected children and the total number of patients inevitably increased daily despite schools' adherence to government and Ministry of Public Health policies.

Previous studies have reported flare-ups or worsening autoimmune diseases following exposure to COVID-19 infection or vaccination (12–15). The flare rate ranged between 0.4% and 20%, and flare symptoms included arthritis, skin rash, fever, leukopenia, thrombocytopenia, neuropathy, and weakness (16–26). Possible mechanisms of vaccine-induced autoimmunity include molecular mimicry, epitope spreading, bystander activation, and polyclonal activation (27–30). Vaccine components, including adjuvants and preservatives, can also activate the immune response (29). Each type of vaccination can produce side effects through different mechanisms. The mRNA and adenovirus-based vaccines can trigger pattern-recognition receptors, subsequently activating intracellular signaling cascades. This process results in inflammasome activation and the production of type I interferon (31). A previous study discovered that the mRNA vaccine transiently increased DNA damage accumulation, type I interferon expression, and oxidative stress in healthy controls. In contrast, patients with systemic lupus erythematosus (SLE) exhibited persistent increases in type I interferon expression and oxidative stress due to the impairment of nucleotide excision repair pathways (32). This mechanism can lead to autoantibody production and the worsening of SLE disease.

COVID infection further affects patients with autoimmune diseases. Severe acute respiratory syndrome coronavirus (SARS-CoV-2) shares similar structures with self-antigen. Therefore, SARS-CoV-2 can disrupt immunologic tolerance and cause cross-reactivity through a molecular mimicry mechanism (33). Previous studies reported that 33 human peptides cross-reacted with SARS-CoV-2 (34) and that numerous immunoreactive viral epitopes and human proteins are shared (35). These findings support the idea that SARS-CoV-2 may induce cross-reactivity and cause autoimmune conditions. In addition, a previous study reported that only 5 of 988 COVID-19-infected patients had juvenile idiopathic arthritis (JIA) and flare symptoms up to 4 weeks after infection (14). Given the concern about the safety of COVID-19 vaccination in children with rheumatic diseases and the limited data in Southeast Asia on the effect of COVID-19 infection during the Omicron variant phase of the pandemic, we aimed to determine the outcomes in children with rheumatic diseases following COVID-19 vaccination and infection when the Omicron variant was the predominant strain in Thailand.

## 2. Materials and methods

### 2.1. Patients and data collection

This retrospective study was performed at two large academic centers in Thailand. Study candidates were patients aged <18 years before diagnosis with rheumatic disease who had regular follow-ups before November 2022 at the Pediatric Rheumatology Clinic at the Faculty of Medicine Ramathibodi Hospital and the Faculty of Medicine Siriraj Hospital, and the Pediatric Nephrology Clinic at the Faculty of Medicine Ramathibodi Hospital, Mahidol University, Bangkok, Thailand. During the COVID-19 pandemic, all patients were routinely queried about their COVID-19 infection and vaccination histories. Patients with rheumatic disease who received at least one dose of the COVID-19 vaccine or had a history of COVID-19 infection with more than 6 months of follow-up time after the last vaccine dose or COVID-19 infection were included in the study. COVID-19 infection in this study was confirmed by SARS-CoV-2 positivity using polymerase chain reaction testing, rapid antigen testing, or both. The following information was extracted from medical records: demographic data, underlying rheumatic diseases, obesity, method of SARS-CoV-2 testing, number and type of COVID-19 vaccines, symptoms, clinical course and outcomes of COVID-19 vaccination and infection, treatment of COVID-19 infection and underlying disease, medications at the time of vaccination and infection, and disease activity before and after COVID-19 vaccination and infection. Body mass index (BMI) was computed as weight (kg) divided by height squared (m^2^) and reported as a Z-score using World Health Organization Anthro software for children aged less than 5 years (36) and AnthroPlus software for children aged 5–19 years (37). The ethics committees of the Faculty of Medicine Ramathibodi Hospital and the Faculty of Medicine Siriraj Hospital approved this multicenter research study (COA. MURA2022/695), which was conducted under the Declaration of Helsinki. The ethical committees of the institutions waived the requirement for informed consent.

### 2.2. Disease activity and outcome measures

During the follow-up visit, the Juvenile Arthritis Disease Activity Score-27 (JADAS-27) (38) was used to assess disease activity in patients with JIA. We used the SLE Disease Activity Index 2000 (SLEDAI-2K) (39) to measure SLE disease activity. We used disease-specific criteria to define inactive disease status: Wallace's criteria for JIA (40), the clinical SLEDAI-2K (cSLEDAI-2K) for SLE, excluding anti-double-stranded DNA and complement, and equal to zero regardless of medication (41), Pediatric Rheumatology International Trials Organization criteria for juvenile dermatomyositis (JDM) (42), and the Pediatric Vasculitis Activity Score for primary systemic vasculitis (43, 44). In other rheumatic diseases, the treating expert clinician defined the inactive condition. Remission status was defined as inactive status for at least 1 year regardless of medication.

Flare after COVID-19 vaccination or COVID-19 infection was defined as a flare that occurred within 3 months of receiving any dose of COVID-19 vaccination or having COVID-19 infection with no other flare-related factors. A flare was diagnosed if patients had new or worsening signs of underlying rheumatic disease without de-escalating concurrent medication. Flare severity was described as follows: (1) mild, self-limiting, or did not require escalation of medication; (2) mild to moderate, requiring an increase in the dosage of corticosteroids or immunosuppressants; and (3) severe, requiring an increase in dosages of both corticosteroids and immunosuppressants or biological agents. In our centers, we routinely adjusted medication after COVID-19 immunization during non-active status in accordance with The American College of Rheumatology recommendation (45). However, patients who did not receive the COVID-19 vaccine from our hospital may not have adjusted their medications after receiving the COVID-19 vaccination.

### 2.3. Statistical analysis

We used IBM SPSS statistics 25 software (IBM Corp., Armonk, NY, USA) and GraphPad Prism 8.3 software (GraphPad Software Inc., La Jolla, California, USA) for data analyses. Continuous variables were presented as mean and standard deviation and categorical variables were presented as numbers and percentages. Non-normally distributed variables were expressed as the median and interquartile range (IQR). The Student *t*-test (unpaired data) or pair *t*-test (paired data) was used to compare two groups with continuous data. Categorical data were compared using the *χ*^2^ test or Fisher exact test. The probabilities of flare and inactive disease after flare from COVID-19 vaccination were analyzed using the Kaplan–Meier method with the log-rank test. Time to flare using the Kaplan–Meier method was indicated from the last dose of COVID-19 vaccination before symptoms of a flare. Predictive factors of flare and inactive disease after flare from COVID-19 vaccination were analyzed using Cox proportional hazards regression analysis, and the hazard ratio (HR) and confidence interval (CI) were provided. For statistical significance, a two-sided *p*-value of less than 0.05 was accepted.

## 3. Results

### 3.1. Patient characteristics

A total of 479 patients with rheumatic diseases were included in the study. Around half of the patients had JIA, which the majority of these patients having enthesitis-related arthritis (ERA), followed by systemic JIA, oligoarticular (oligo) JIA, and polyarticular JIA with rheumatoid factor (RF) positive (poly-RF-pos) and negative (poly-RF-neg). Around 40% of the participants in this study had connective tissue diseases (CTDs), the majority of whom had SLE, followed by JDM and overlapping syndromes. Nearly 10% of patients had vasculitis, with IgA vasculitis being the most prevalent, followed by Takayasu arteritis. Additional details are presented in Supplementary Table S1. From a total, 315 (65.76%) were female, with a mean age of 13.81 ± 4.30 years. The mean disease duration was 4.84 ± 4.22 years. The mean dose of prednisolone was highest in patients with CTDs. Hydroxychloroquine (HCQ), azathioprine, mycophenolate mofetil, and intravenous cyclophosphamide were frequently used in these patients. Patients with JIA typically received methotrexate (MTX), sulfasalazine, and leflunomide. Most patients with rheumatic diseases in this study did not receive biological agents. Only 65 (13.57%) of patients received biological agents. The most frequently used biological agents were tumor necrosis factor-alpha inhibitors, including adalimumab, etanercept, and infliximab. Of 479 patients included, 32 (6.68%) did not receive COVID-19 vaccination. Four hundred and twenty-nine (89.56%) patients received COVID-19 vaccines after the diagnosis of rheumatic disease, whereas 15 (3.13%) received COVID-19 vaccines within 3 months of their last dose before the diagnosis of rheumatic disease. Only 3 (0.63%) patients received COVID-19 vaccines more than 6 months after their last dose before the diagnosis of rheumatic diseases. Of 479 patients, 230 (48.01%) had no history of COVID-19 infection. Of 249 patients with confirmed COVID-19 infection, 245 (98.39%) had COVID-19 infection after the diagnosis of rheumatic diseases, and 4 (1.61%) had COVID-19 infection before the onset of rheumatic diseases. Most patients received the mRNA vaccines and two doses of COVID-19 vaccination. Additional information regarding the patient characteristics of this study is shown in Table 1.

**Table 1:** Characteristics of children with rheumatic diseases included in the study (*n* = 479).

Characteristics	JIA (*n* = 229)	CTDs (*n* = 189)	Vasculitis (*n* = 42)	Others (*n* = 19)
Age, years	13.56 ± 4.23	14.90 ± 3.98	11.74 ± 4.53	10.50 ± 4.27
Female sex, *n* (%)	120 (52.40)	156 (82.54)	24 (57.14)	15 (78.94)
Disease duration, years	5.31 ± 3.79	4.51 ± 3.61	4.26 ± 3.02	3.61 ± 2.81
**Medications, *n* (%)**				
Prednisolone dose, mg/day	1.78 ± 0.40	6.87 ± 0.60	1.17 ± 0.51	3.83 ± 1.74
Hydroxychloroquine	14 (6.11)	154 (81.48)	0 (0)	0 (0)
Methotrexate	134 (58.52)	17 (8.99)	7 (16.67)	8 (42.11)
Sulfasalazine	70 (30.57)	0 (0)	0 (0)	0 (0)
Azathioprine	6 (2.62)	63 (33.33)	4 (9.52)	2 (10.53)
MMF	2 (0.87)	24 (12.70)	0 (0)	1 (5.26)
Cyclosporin	2 (0.87)	8 (4.23)	0 (0)	1 (5.26)
IVCY	0 (0)	29 (15.34)	0 (0)	1 (5.26)
Leflunomide	15 (6.55)	1 (0.53)	0 (0)	0 (0)
Tacrolimus	0 (0)	2 (1.06)	0 (0)	0 (0)
**Biological agents, *n* (%)**				
Etanercept	21 (9.17)	0 (0)	0 (0)	0 (0)
Adalimumab	25 (10.92)	0 (0)	0 (0)	3 (15.79)
Infliximab	1 (0.44)	0 (0)	0 (0)	0 (0)
Tocilizumab	13 (5.68)	0 (0)	1 (2.38)	0 (0)
Rituximab	0 (0)	1 (0.53)	0 (0)	0 (0)
**Total doses of COVID-19 vaccination, *n* (%)** ^a^				
None	16 (6.99)	5 (2.65)	9 (21.43)	2 (10.53)
1 dose	17 (7.42)	8 (4.23)	0 (0)	2 (10.53)
2 doses	119 (51.97)	91 (48.15)	23 (54.76)	12 (63.16)
3 doses	60 (26.20)	81 (42.86)	10 (23.81)	2 (10.53)
4 doses	15 (6.55)	4 (2.12)	0 (0)	1 (5.26)
5 doses	2 (0.87)	0 (0)	0 (0)	0 (0)
**Vaccination type, *n* (%)**				
Inactive	3 (1.31)	2 (1.06)	0 (0)	1 (5.26)
Adenovirus vector	1 (0.44)	0 (0)	0 (0)	0 (0)
mRNA	172 (75.11)	154 (81.48)	29 (69.05)	15 (78.95)
Mixed	37 (16.16)	28 (31.46)	4 (0.52)	1 (5.26)
**Vaccine information**				
Not received	16 (6.99)	5 (2.65)	9 (21.43)	2 (10.53)
Received while having rheumatic disease	205 (89.52)	176 (93.12)	32 (76.19)	16 (84.21)
New onset of rheumatic disease within 3 months of vaccination	8 (3.49)	6 (3.17)	0 (0)	1 (5.26)
New onset of rheumatic disease within 6 months of vaccination	0 (0)	2 (1.06)	1 (2.38)	0 (0)
**COVID-19 infection**				
No history of infection	118 (51.53)	90 (47.62)	15 (35.71)	7 (36.84)
Infection while having a rheumatic disease	109 (47.60)	97 (51.32)	27 (64.29)	12 (63.16)
New onset of rheumatic disease within 3 months of infection	2 (0.90)	2 (1.06)	0 (0)	0 (0)
**Severity of infection**				
- Mild	107 (98.17)	96 (98.97)	27 (100)	12 (100)
- Moderate	2 (1.83)	1 (1.03)	0 (0)	0 (0)
- Severe	0 (0)	0 (0)	0 (0)	0 (0)

Data are presented as mean ± standard deviation. JIA, juvenile idiopathic arthritis; CTDs, connective tissue diseases; IVCY, intravenous cyclophosphamide; MMF, mycophenolate mofetil; COVID-19, coronavirus disease 2019.

^a^ The total number of COVID-19 vaccinations that each patient had received.

### 3.2. COVID-19 vaccine-related outcomes in rheumatic diseases

Among 429 patients who received at least one dose of COVID-19 vaccination after having rheumatic diseases, most patients did not have side effects, with around 12% developing a fever and only 5% having pain at the injection site. The details of the side effects of the COVID-19 vaccination in each disease type are provided in Supplementary Table S2. There was no statistical difference between vaccine-associated side effects on the type of disease (*p* = 0.970) and the treatment (high-dose vs. low-dose prednisolone; *p* = 0.091). After receiving COVID-19 vaccination, 46 (10.72%) patients developed a disease flare-up (Table 2). Rheumatic diseases in patients with flare included JIA (24; 52.17%), including 2 (8.33%) patients with OligoJIA, 2 (8.33%) with poly-RF-neg, 3 (12.50%) with poly-RF-pos, 12 (50.00%) with ERA, 4 (16.67%) with systemic JIA, and 1 (4.17%) with psoriatic arthritis. Nineteen (41.30%) patients with CTDs had flare, including 17 (89.47%) with SLE and 2 (10.53%) with JDM. Two (4.35%) patients with vasculitis developed a Behçet's disease (BD) flare-up, and one (2.17%) patient had immune-mediated necrotizing myopathy.

**Table 2:** Characteristics of patients who had received at least one dose against COVID-19 (*n* = 429).

Characteristics	Flare (*n* = 46)	Non-flare (*n* = 383)	*p*-value
**Underlying rheumatic diseases, *n* (%)**			
JIA	24 (52.17)	181 (47.26)	0.88
CTDs	19 (41.30)	157 (40.99)	
Vasculitis	2 (4.35)	30 (7.83)	
Other	1 (2.17)	15 (3.92)	
**Demographic data**			
Female, *n* (%)	29 (63.04)	257 (67.10)	0.62
Age, years	14.37 ± 3.89	14.15 ± 4.14	0.73
Disease duration, years	4.14 ± 3.06	5.02 ± 3.71	0.12
Obesity, *n* (%)	7 (15.22)	51 (13.32)	0.91
BMI Z-score	0.26 ± 1.61	0.53 ± 1.55	0.30
**Medications, *n* (%)**			
Prednisolone dose, mg/day	6.63 ± 1.49	3.57 ± 0.34	0.006^a^
Hydroxychloroquine	19 (41.30)	137 (35.77)	0.517
Methotrexate	20 (43.48)	130 (33.94)	0.252
Sulfasalazine	15 (32.61)	52 (13.58)	0.002^a^
Azathioprine	1 (2.17)	7 (1.83)	0.05
MMF	4 (8.70)	20 (5.22)	0.31
Cyclosporin	2 (4.35)	5 (1.31)	0.17
IVCY	1 (2.17)	28 (7.31)	0.345
Leflunomide	1 (2.17)	13 (3.39)	1.00
Tacrolimus	0 (0)	2 (0.52)	1.00
**Biological agents, *n* (%)**			
Etanercept	2 (4.35)	19 (4.96)	0.169
Adalimumab	4 (8.70)	21 (5.48)	
Infliximab	1 (2.17)	0 (0)	
Tocilizumab	2 (4.35)	10 (2.61)	
Rituximab	0 (0)	1 (0.26)	
**Disease status before vaccination, *n* (%)**			
Active	20 (43.48)	100 (26.11)	0.0002^a^
Inactive	24 (52.17)	154 (40.21)	
Remission	1 (4.35)	129 (33.68)	
**Total doses of COVID-19 vaccination, *n* (%)** ^b^			
1 dose	5 (10.87)	17 (4.44)	0.11
2 doses	19 (41.30)	216 (56.40)	
3 doses	21 (45.65)	130 (33.94)	
4 doses	1 (2.17)	18 (4.70)	
5 doses	0 (0)	2 (0.52)	
**Vaccination type, *n* (%)**			
Inactive	0 (0)	6 (1.57)	0.36
Adenovirus vector	0 (0)	1 (0.26)	
mRNA	42 (91.30)	310 (80.94)	
Mixed	4 (8.70)	66 (17.23)	
Adjust medication after vaccination, *n* (%)	6 (13.04)	52 (13.58)	1.00
**Disease activity scores before and after vaccination**			
JADAS-27 before vaccination	4.74 ± 4.89	4.09 ± 5.14	0.57
SLEDAI-2K before vaccination	1.47 ± 1.74	1.54 ± 2.45	0.91
JADAS-27 after vaccination	5.95 ± 6.02	2.81 ± 4.11	0.001^a^
SLEDAI-2K after vaccination	2.65 ± 2.83	1.09 ± 1.72	0.002^a^

Data are presented as mean ± standard deviation. JIA, juvenile idiopathic arthritis; CTDs, connective tissue diseases; BMI, body mass index; IVCY, intravenous cyclophosphamide; MMF, mycophenolate mofetil; JADAS, arthritis disease activity score; SLEDAI-2K, systemic lupus erythematosus disease activity index 2000; COVID-19, coronavirus disease 2019.

^a^ A value of *p* < 0.05 was considered to indicate statistical significance.

^b^ The total number of COVID-19 vaccinations that each patient had received.

**Figure 1:**
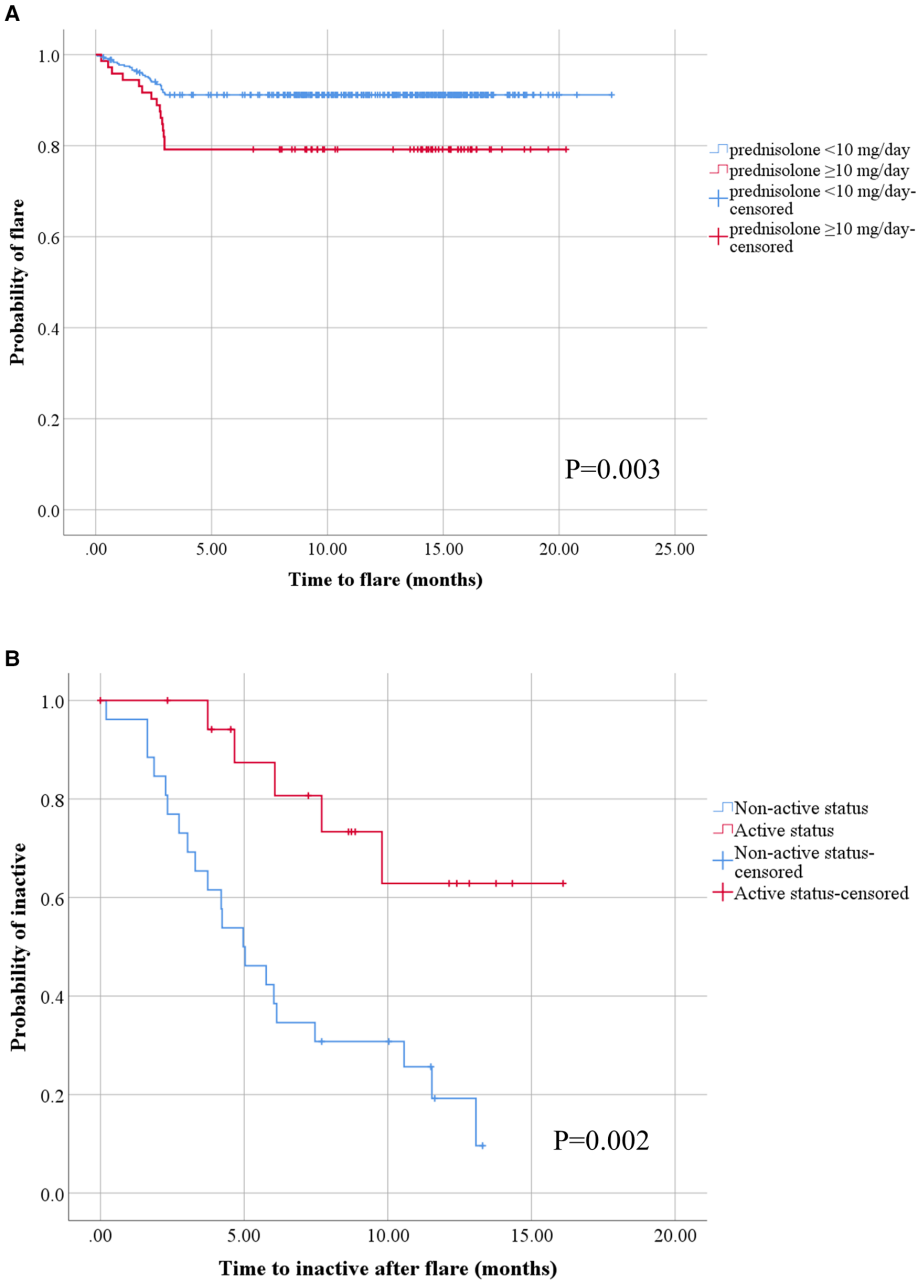
Kaplan–Meier analysis of disease flare and attainment of inactive status after disease flare in children with rheumatic diseases following COVID-19 vaccination. All patients with at least one dose of COVID-19 vaccination after having rheumatic diseases (**A**), the blue line represents patients with prednisolone <10 mg/day group (*n* = 356) and the red line represents patients with prednisolone ≥10 mg/day group (*n* = 73). All patients with disease flare after COVID-19 vaccination (**B**), the blue line represents patients with non-active status (inactive and remission) before COVID-19 vaccination (*n* = 26) and the red line represents patients with active status before COVID-19 vaccination (*n* = 20).

When we classified patients into flare and non-flare groups, we observed that age, sex, BMI Z-scores, and disease duration before receiving the first COVID-19 vaccination were comparable across the two groups. Substantially distinct frequencies of disease status were observed in the flare and non-flare groups before the first dose of COVID-19 vaccination was received. The vaccine type was the mRNA vaccine, and patients frequently received two to three vaccine doses. Most side effects occurred after receiving the first dose of COVID-19 immunization, and fever was the most common side effect in both groups. The dose of prednisolone at the time of vaccination was substantially higher in the flare group than in the non-flare group (Table 2). The flare group more frequently received sulfasalazine than the non-flare group. The use of other medications (disease-modifying antirheumatic drugs and biological agents) was comparable between the two groups. JADAS-27 and SLEDAI-2K scores before vaccination were similar between patients with flare and non-flare. However, JADAS-27 and SLEDAI-2K scores after vaccination significantly increased in the flare group compared with those in the non-flare group (Table 2). At the end of the study, the mean time to flare after COVID-19 vaccination based on the Kaplan–Meier method was longer in patients receiving prednisolone <10 mg/day compared with that in patients receiving prednisolone ≥10 mg/day (20.47 months, 95% CI: 19.86–21.07 vs. 16.51 months, 95% CI: 14.80–18.22, *p* = 0.003; Figure 1). Univariate analysis identified two significant covariates: use of prednisolone ≥10 mg/day before vaccination and non-active status (i.e., inactive disease and remission status). After multivariate analysis adjusted for age, sex, obesity, disease duration, disease status, and treatment, the only predictor of flare following the COVID-19 vaccine was the use of prednisolone ≥10 mg/day before vaccination (HR: 2.04, 95% CI: 1.05–3.97, *p* = 0.037), as shown in Table 3.

**Table 3:** Predictors of disease flare following COVID-19 vaccination in children with rheumatic diseases.

Predictive factors	Univariate analysis			Multivariate analysis		
	HR	95% CI	*p*-value	HR	95% CI	*p*-value
Age	0.99	0.92–1.07	0.85			
Sex	1.19	0.65–2.16	0.57			
Receiving prednisolone ≥10 mg/day	2.45	1.32–4.54	0.004^a^	2.04	1.05–3.97	0.037^a^
Active disease before vaccination	2.07	1.14–3.73	0.016^a^	1.66	0.87–3.13	0.12
Obesity	1.10	0.53–2.28	0.80			
Disease duration ≤2 years	1.46	0.75–2.84	0.27			
Biologic agent	1.54	0.74–3.19	0.24			
Adjust medication	0.91	0.39–2.16	0.84			

HR, hazard ratio; CI, confidence interval; COVID-19, coronavirus disease 2019.

Data were analyzed by Cox proportional hazards regression analysis.

^a^ A value of *p* < 0.05 was considered to indicate statistical significance.

In the flare group, the mean time to flare after the last dose of COVID-19 vaccination and before flare symptoms was 1.95 ± 0.14 months. Of 46 patients, flare occurred predominantly after the second vaccine dose (26; 56.52%), followed by the first (11; 23.91%) and third (9; 19.57%) doses. The features of flare were as follows: arthritis (52.17%), thrombocytopenia (13.00%), anemia (6.52%), inflammatory or vasculitis rash (10.87%), progressive weakness (2.17%), hepatitis (2.17%), and systemic inflammation (10.87%). Flare severity was mostly mild to moderate (52.17%), followed by mild (26.09%) and severe (21.74%). For the treatment of flares, patients mostly required increased doses of the following therapies: immunosuppressants other than corticosteroids (34.78%), corticosteroids (17.39%), both corticosteroids and immunosuppressants (13.04%), and biologic agents (8.70%). Of 46 flare patients, 26 (56.52%) achieved inactive clinical status after disease flares in a mean time of 5.60 ± 3.15 months. At the end of the study, the mean time to reach clinically inactive disease after disease flare using the Kaplan–Meier method was longer in the patients with active disease before receiving the COVID-19 vaccine than in patients with inactive disease status (12.65 months, 95% CI: 10.18–15.13 vs. 6.34 months, 95% CI: 4.70–8.06, *p* = 0.002; Figure 1). We conducted a multivariate analysis by selecting significant covariates in the univariate analysis. After adjusting for age, sex, obesity, disease duration, disease status, and treatment, the analysis showed that the predictor of achieving inactive status after disease flare was non-active disease before receiving COVID-19 vaccination (HR: 2.95, 95% CI: 1.04–8.40, *p* = 0.043; Table 4).

**Table 4:** Predictors of inactive disease after disease flare following COVID-19 vaccination.

Predictive factors	Univariate analysis			Multivariate analysis		
	HR	95% CI	*p*-value	HR	95% CI	*p*-value
Age	1.04	0.94–1.15	0.49			
Sex	1.54	0.70–3.36	0.28			
Receiving prednisolone <10 mg/day	4.46	1.34–14.86	0.015^a^	2.68	0.74–9.73	0.13
Non-active disease before vaccination^b^	4.25	1.60–11.30	0.004^a^	2.95	1.04–8.40	0.043^a^
Obesity	1.08	0.37–3.18	0.89			
Disease duration ≤2 years	1.31	0.55–3.13	0.54			
Biologic agent	1.09	0.41–2.89	0.87			
Adjust medication	1.12	0.38–3.30	0.83			

HR, hazard ratio; CI, confidence interval; COVID-19, coronavirus disease 2019.

Data were analyzed by Cox proportional hazards regression analysis.

^a^ A value of *p* < 0.05 was considered to indicate statistical significance.

^b^ Non-active disease includes patients with inactive status and those in remission.

### 3.3. New-onset rheumatic diseases following the COVID-19 vaccine

Of 447 patients who received at least one dose of COVID-19 vaccination, 15 (3.36%) developed a new onset of rheumatic disease after receiving the vaccine. Most were patients with JIA [8; 53.33%; including ERA (4; 50.00%), poly-RF-pos (2; 25%), poly-RF-neg (1; 12.5%), and OligoJIA (1; 12.5%)], followed by CTDs patients [6; 40.00%; including SLE (4; 66.67%), JDM (1; 16.67%), Sjögren's syndrome (1; 16.67%)], and one (6.67%) patient with neuromyelitis optica (i.e., other rheumatic diseases). All patients received the mRNA vaccine BNT162b2. Of 15 patients, 10 (66.67%) and 5 (33.33%) developed a new onset of rheumatic disease after the second and first COVID-19 vaccine doses, respectively. Three patients had fevers after receiving the first dose of the COVID-19 vaccination. The median time from the last COVID-19 vaccination to the new onset of disease was 2.50 (1.73–2.77) months. The median patient age was 11.50 years (IQR: 10.34–13.72). Two-thirds of patients were female and 26.67% were obese, and the median Z-score was 0.45 (IQR: −0.66–2.23).

### 3.4. COVID-19 infection-related outcomes in rheumatic diseases

Of 245 patients who had COVID-19 infection, 8 (3.27%) had a disease flare after COVID-19 infection (Table 5). No patients with rheumatic diseases in this study developed multisystem inflammatory syndrome in children after COVID-19 infection. When patients were classified into the flare or non-flare groups, most patients in the flare group had SLE (4; 50%), followed by JIA (2; 25%; one with systemic JIA and one with poly-RF-pos), and vasculitis (2; 25%; one with microscopic polyangiitis and one with IgA vasculitis). Age, the percentage of female patients, and BMI Z-score were comparable between the flare and non-flare groups. Most patients in the flare and non-flare groups had a similar disease status before the COVID-19 infection. JADAS-27 and SLEDAI-2K scores before infection were comparable between patients with flare and non-flare. Nevertheless, the JADAS-27 and SLEDAI-2K scores after infection were significantly higher in the flare group compared with those in the non-flare group (Table 5). The predominant vaccine type was the mRNA vaccine, and patients frequently received two doses of COVID-19 vaccines. Disease duration before COVID-19 infection was similar between the two groups. The amount of prednisolone during COVID-19 infection and the proportion of patients receiving immunosuppressants other than AZA were comparable between the two groups. In the flare group, no patients received biological agents. The symptoms of COVID-19 infection in both groups were fever, cough, and sore throat. The most frequent anti-viral drug was favipiravir, and patients did not require hospital admission. All patients recovered after COVID-19 infection, which lasted fewer than five days based on COVID-19 symptoms. No significant parameters—including doses of prednisolone and disease status before COVID-19 infection—were associated with flares following COVID-19 infection.

**Table 5:** Characteristics of children with rheumatic diseases and COVID-19 infection (*n* = 245).

Characteristics	Flare (*n* = 8)	Non-flare (*n* = 237)	*p*-value
**Underlying rheumatic diseases, *n* (%)**			
JIA	2 (25)	107 (45.15)	0.36
CTDs	4 (50)	93 (39.24)	
Vasculitis	2 (25)	25 (10.55)	
Others	0 (0)	12 (5.06)	
**Demographic data**			
Female, *n* (%)	6 (75)	164 (69.20)	1.00
Age, years	14.47 ± 4.09	13.28 ± 8.90	0.71
Disease duration, years	4.32 ± 3.46	4.78 ± 3.78	0.73
Obesity, *n* (%)	2 (25)	36 (15.25)	0.65
BMI Z-score	0.56 ± 134	0.51 ± 1.59	0.93
**Medications, *n* (%)**			
Prednisolone dose, mg/day	4.06 ± 4.21	3.70 ± 7.24	0.89
Hydroxychloroquine	3 (37.5)	75 (31.65)	0.71
Methotrexate	1 (12.5)	93 (39.24)	0.16
Sulfasalazine	1 (12.5)	32 (13.50)	1.00
Azathioprine	4 (50)	35 (14.77)	0.025^a^
MMF	0 (0)	12 (5.06)	1.00
Cyclosporin	0 (0)	6 (2.53)	1.00
IVCY	1 (12.5)	8 (3.38)	0.26
Leflunomide	0 (0)	9 (3.80)	1.00
Tacrolimus	0 (0)	1 (0.42)	1.00
**Biological agents, *n* (%)**			
Etanercept	0 (0)	13 (5.49)	1.00
Adalimumab	0 (0)	12 (5.06)	
Infliximab	0 (0)	1 (0.42)	
Tocilizumab	0 (0)	6 (2.53)	
**Disease status before infection, *n* (%)**			
Active	2 (25)	78 (32.91)	0.89
Inactive	4 (50)	102 (43.04)	
Remission	2 (25)	57 (24.05)	
**Total doses of COVID-19 vaccination** ^b^			
None	2 (25)	28 (11.81)	0.58
1 dose	1 (12.5)	17 (7.17)	
2 doses	4 (50)	102 (43.04)	
3 doses	1 (12.5)	80 (33.76)	
4 doses	0 (0)	10 (4.22)	
**Vaccination type, *n* (%)**			
Inactive	0 (0)	4 (1.69)	0.92
mRNA	5 (62.5)	163 (68.78)	
Mixed	1 (12.5)	42 (17.72)	
**Disease activity scores before and after infection**			
JADAS-27 before infection	5 ± 2.83	4.58 ± 5.98	0.92
SLEDAI-2K before infection	0.67 ± 1.16	1.56 ± 2.37	0.52
JADAS-27 after infection	12.50 ± 2.12	3.85 ± 5.58	0.031^a^
SLEDAI-2K after infection	6.5 ± 4.44	1.63 ± 2.49	0.001^a^
**Symptoms of COVID-19 infection, *n* (%)**			
Fever	6 (75)	172 (72.57)	1.00
Cough	6 (75)	146 (61.60)	0.71
Sore throat	4 (50)	123 (51.90)	1.00
Nasal congestion	6 (75)	124 (52.32)	0.29
Headache	2 (25)	17 (7.17)	0.12
Pneumonia	0 (0)	3 (1.27)	1.00
Diarrhea/vomiting	0 (0)	12 (5.06)	1.00
**Treatment for COVID-19 infection, *n* (%)**			
Favipiravir	2 (25)	110 (46.41)	0.29
Monopiravir	0 (0)	8 (3.38)	1.00
Herb (andrographolide)	2 (25)	31 (13.08)	0.29
Oxygen therapy	0 (0)	3 (1.27)	1.00
Ward admission	0 (0)	13 (5.49)	1.00
Home isolation	8 (100)	224 (94.51)	1.00
**Outcome of COVID-19 infection, *n* (%)**			
Recovery	8 (100)	232 (97.89)	1.00
Long COVID	0 (0)	5 (2.11)	
Day of recovery^c^	4.50 ± 2.93	4.42 ± 2.52	0.93

Data are presented as mean ± standard deviation. JIA, juvenile idiopathic arthritis; CTDs, connective tissue diseases; BMI, body mass index; IVCY, intravenous cyclophosphamide; MMF, mycophenolate mofetil; JADAS, juvenile arthritis disease activity score; SLEDAI-2K, systemic lupus erythematosus disease activity index 2000; COVID-19, coronavirus disease 2019.

^a^ A value of *p* < 0.05 was considered to indicate statistical significance.

^b^ The total number of COVID-19 vaccinations that each patient had received.

^c^ Day of recovery means duration of patients recovered from COVID-19 infection based on clinical symptoms.

In the flare group, the mean time to flare following COVID-19 infection was 1.13 ± 0.94 months. The features of flares included glomerulonephritis (3; 37.5%), inflammatory or vasculitis rash (2; 25%), arthritis (2; 25%), and autoimmune hemolytic anemia (1; 12.5%). Flare severity was mainly mild to moderate (62.5%), followed by severe (37.5%). In the treatment of the underlying rheumatic disease after the flare, patients primarily required increased doses of the following therapies: immunosuppressants other than corticosteroids (50%), corticosteroids and immunosuppressants (37.5%), and corticosteroids alone (12.5%). Of 8 patients, 5 (62.5%) achieved inactive disease status with a mean duration of 4.26 ± 2.41 months.

Regarding the COVID-19 course, there was no statistical difference between the specific diseases (Supplementary Table S3) and the treatment, including patients receiving high-dose and low-dose prednisolone (Supplementary Table S4) and patients with and without biological treatment (Supplementary Table S5). In addition, the course of COVID-19 infection in patients who developed an infection after vaccination was comparable to that in patients who had COVID-19 infection prior to vaccination (Supplementary Table S6). Most patients had a mild infection with only upper respiratory tract symptoms and recovery within five days. Only three COVID-19 patients had a moderate infection with mild pneumonia and needed only an oxygen canular. All of them were treated with favipiravir. They had already received the vaccination, and none of them had a disease flare. The first case was an inactive SLE who had taken only HCQ. Her recovery time took around 2 weeks. The second case was inactive SJIA, who had taken low-dose prednisolone and MTX. She recovered within 10 days. The third case is inactive oligo JIA, who had taken MTX. Her recovery time was five days.

### 3.5. New-onset rheumatic diseases following COVID-19 infection

Of 249 patients who had confirmed COVID-19 infection, 4 (1.61%) developed a new onset of rheumatic disease—including ERA, OligoJIA, JDM, and SLE—after having COVID-19 illness. Two patients (50.00%) did not receive a COVID-19 vaccination. The other patients received two doses of BNT162b2; the last dose of the COVID-19 vaccine was received more than 6 months before the onset of symptoms of new rheumatic disease. The mean time between the onset of symptoms of new rheumatic diseases and COVID-19 infection was 2.59 ± 0.23 months. The mean patient age was 11.86 ± 1.65 years, with a similar representation of male and female patients. All patients had normal weight and the mean Z-score was 1.11 ± 0.98. The symptoms of COVID-19 infection of the four patients included fever (100%), cough (50%), sore throat (50%), nasal congestion (25%), and diarrhea (25%). No patient required hospital admission during COVID-19 illness. Two patients received favipiravir, and one patient received herbal medicine. Symptoms of COVID-19 infection resolved for all patients within 5 days of infection.

## 4. Discussion

We observed that approximately 90% of patients with rheumatic disease received at least two doses of COVID-19 vaccination, and 10% developed disease flares, which were mostly mild to moderate. Roughly 70% of patients with disease flares after COVID-19 vaccination required escalated treatment for their underlying rheumatic conditions. Use of prednisolone ≥10 mg/day was associated with disease flare after COVID-19 vaccination. During the Omicron-predominant phase of the pandemic, approximately half of the patients with rheumatism in our study had COVID-19 infection with mild symptoms, and 3% had a disease flare. However, all patients with disease flare after COVID-19 infection required increased treatment for their underlying rheumatic diseases. The frequency of patients with new-onset autoimmune disease after COVID-19 vaccination and infection was fairly low.

The infection and mortality rates of COVID-19 have varied worldwide (1). Many factors, including the change in the epidemiology of COVID-19 variants over time and varying COVID-19 strategies and vaccination campaigns in each country, contribute to these variations. In addition, the variability in flare rates in rheumatic disease across studies was affected by the number of participants, age, the type of vaccine, COVID-19 strains, the type of rheumatic disease, disease status, and medications. A study of 36 adolescents with rheumatic disease from the European League Against Rheumatism (EULAR) Coronavirus vaccine (COVAX) physician-reported registry showed that only one patient had mild polyarthralgia, suggesting reactogenicity rather than true disease flare, and two of 74 adults with JIA had mild polyarthralgia and uveitis (46). This study showed a lower frequency (2.70%) of disease flares compared with our study (10.27%); this finding may be explained by the fact that most patients in the EULAR COVAX physician-reported registry were in remission. A low flare rate was also reported in a Singapore study, which found a 4.4% flare rate in 159 patients after the second mRNA vaccine (47). Rheumatic patients with inactive or low disease activity were recruited into the Singapore study, whereas nearly half of our patients were still in active disease before receiving COVID-19 vaccination. This may explain the higher flare rate in our study compared with that in previous studies. The higher rate we observed is in line with previous evidence that 18% of adult rheumatic patients in their cohort had a flare, and most patients in the flare group had active disease (13). This evidence further supported that patients with active disease before receiving COVID-19 vaccination had a higher risk of flare (HR: 1.4, 95% CI: 1.2–1.6) (13). Furthermore, our study showed that the use of prednisolone doses ≥10 mg/day at the time of vaccination was a risk factor for flares, as it might indicate that the underlying disease in study patients who received prednisolone doses ≥10 mg/day may not have been truly quiescent despite the lack of active symptoms. The risk of flare-ups after vaccination in previous studies (16, 19, 24) was also associated with a history of disease flare within 6 months of receiving the COVID-19 vaccine, similarly suggesting that the underlying rheumatic disease was not entirely inactive. Consistent with findings from previous studies (13, 16, 18, 20), most patients in our study experienced a mild-to-moderate flare with arthritis as the most prevalent symptom. However, some studies reported no disease flare following COVID-19 vaccination; this finding may be attributable to the small number of study participants (48), or that flares, which were self-reported by patients via the telephone, were underreported (12). We did not identify other factors—including the type of vaccination and other immunosuppressive medications—that were associated with disease flare. Because most of our patients received an mRNA vaccine, we could not compare the flare rate across types of vaccines. To date, the literature has revealed similar flare rates following mRNA or adenovirus-based vaccinations (30). Our study further demonstrated that flares usually occurred after the second vaccine dose, consistent with findings from previous studies (16, 30). The immune response—particularly antibody production—could reasonably increase substantially after the second vaccination dose, making patients susceptible to autoimmune disease flare-ups. Moreover, patients who had minimal flare-up symptoms in the earlier stage of flare and then later developed ongoing severe symptoms may not have been detected if follow-up was brief. Thus, we intended to closely observe patients at least 3 months after their last vaccine dose.

A previous study reported that patients with inflammatory arthritis had a lower frequency of flares compared with those with systemic rheumatic diseases such as SLE and BD (18). This finding was supported by a Turkish study (25), which reported that a higher frequency of flares was associated with BD and familial Mediterranean fever than with other rheumatic diseases. Another study mentioned that the presence of more than one rheumatic disease was associated with a risk of flare-ups (49). Our study had very few cases of BD and autoinflammatory diseases; therefore, no significant difference was observed in flare-ups after vaccination between the types of rheumatic disease, consistent with findings from previous reports (19, 30).

A previous EULAR recommendation stated that COVID-19 vaccination should be given to patients with inactive rheumatic disease to avoid flare-ups (50). However, immunizing patients with active rheumatic diseases during the COVID-19 pandemic is necessary for preventing severe infection. Therefore, physicians should be aware of flare-ups in those with active disease at the time of COVID-19 vaccination. Duration to the attainment of inactive disease after flare-ups varied between studies. We observed that half of the patients required roughly 6 months to reach inactive disease status. In addition, patients who were inactive or in remission before receiving the COVID-19 vaccine could reach inactive status faster than patients who had active disease before receiving the vaccine. Only one-quarter of study patients had self-limiting symptoms; the remaining patients required increases in the dose of their medications, consistent with findings from the adult study in Singapore (13). Our results indicated that patients often develop flares roughly 2 months after COVID-19 vaccination; therefore, physicians should closely monitor these patients during this period.

The severity of COVID-19 infection varied over time because of the different SAR-CoV-2 variants. Until now, the infection rate of the Omicron variant has been increasing, and the mortality rate has been low. Moreover, infection severity depends on the type and status of rheumatic diseases. A study in early 2020 in China reported that the length of hospital stay and mortality rate in patients with rheumatic diseases were similar to those in patients without rheumatism—except for respiratory failure, which was more common in patients with rheumatic diseases (51). In the study by the European Alliance of Associations for Rheumatology COVID-19 Registry, the Childhood Arthritis and Rheumatology Research Alliance (CARRA) Registry, and the CARRA-sponsored COVID-19 Global Paediatric Rheumatology Database that recruited patients from early 2020 to mid-2021, 43 (7%) of 607 patients were hospitalized. Among these patients, three died, two had active SLE, and one had an auto-inflammatory syndrome. This study reported that SLE, mixed connective tissue disease, vasculitis, autoinflammatory syndrome, and obesity were risk factors for hospitalization (52). During the same period, data from the National Paediatric Rheumatology Database in Germany reported 76 patients with rheumatic diseases and showed that most patients had mild symptoms and had a good recovery. Only two patients were hospitalized, and one died from cardiorespiratory failure. This German database study did not find disease flare following COVID-19 infection (53). In contrast to findings from previous studies, most of the 245 patients with rheumatism and COVID-19 infection in our cohort had mild symptoms, and all recovered. This may be because most infected cases in our study occurred during the Omicron phase of the pandemic and after patients had received COVID-19 vaccination. However, disease flares after COVID-19 infection are still a concern. Only 5 of 988 patients with JIA reported disease flares in the German study. All patients were regularly followed and had inactive disease before infection. The flare developed roughly 3–4 weeks after infection. Arthritis was the only flare symptom in these patients and all patients required additional treatment (14). Only a low incidence of rheumatic disease flare after COVID-19 infection was reported in children (54). This low flare rate was confirmed in our study, which indicated a flare rate of only 3.27%. However, most patients required additional treatment after the flare. The type of rheumatic disease, disease activity, and concurrent medications were not factors associated with flare in our study.

The development of new-onset autoimmune disease after COVID-19 infection and vaccination is also a concern. Besides molecular mimicry, the production of autoantibodies, including ANA, anti-Ro, ANCA, RF, and antiphospholipid antibodies after COVID-19 infection, was reported in several studies (55–58). Moreover, COVID-19 infection can induce immune reactions, including excessive cytokine production, leading to bystander activation (59). As in the pathogenesis of SLE, the production of neutrophil extracellular traps was also reported in patients infected with COVID-19 (60). However, the true incidence of new-onset autoimmune disease is still unknown. One systematic review of the new onset of autoimmune diseases showed that most of these diseases were vasculitis and arthritis, followed by idiopathic inflammatory myopathies and SLE (61). Although a very low incidence of new-onset autoimmune disease was observed in our study, JIA was the most common, followed by SLE; vasculitis was not observed. This finding may be explained by the higher prevalence of patients with SLE in our region than in the European and American reports. Although COVID-19 infection and vaccination may trigger the new onset of autoimmune disease through several mechanisms, the genetic susceptibility of these post-COVID-19 rheumatic patients is a crucial factor (62) that must be explored in further studies.

The main limitations of this study were its retrospective study design and the small number of enrolled patients—especially those with rheumatic disease and confirmed COVID-19 infection. However, we collected routinely available data from two large tertiary-care centers, thus avoiding missing data. Moreover, we routinely assessed the disease activity of patients with rheumatism during their visits, resulting in the collection of JADAS-27 and SLEDAI-2K scores before and following COVID-19 vaccination and infection.

## 5. Conclusion

In our cohort, roughly 10% of patients with rheumatic diseases had disease flare after at least one dose of the COVID-19 vaccine. Flare severity was mostly mild to moderate. Immunization in patients with rheumatic diseases remains highly recommended, given that the benefits of COVID-19 vaccination outweigh the risks. However, pediatric rheumatologists should monitor patients—especially those who receive concurrent prednisolone ≥10 mg/day or have active disease—when administering COVID-19 vaccination. Our study was during the Omicron-predominant phase of the pandemic. Study patients with rheumatic diseases had mild symptoms of COVID-19 illness, and 3% had a disease flare. The frequency of patients with new-onset rheumatic disease following COVID-19 vaccination and infection was low.

## Data Availability

The original contributions presented in the study are included in the article/**Supplementary Material**, further inquiries can be directed to the corresponding author.
