# Supplementary material for: Outcomes in children with rheumatic diseases following COVID-19 vaccination and infection: data from a large two-center cohort study in Thailand
Source: Front Pediatr. 2023 Jun 8;11:1194821. doi: 10.3389/fped.2023.1194821 (PMC10285492; doi:10.3389/fped.2023.1194821)
Supplement: Supplementary file 1 [file Datasheet1.pdf]

Supplementary Material

Outcomes in children with rheumatic diseases following COVID-19 vaccination and infection: Data from a large two-center cohort study in Thailand

Butsabong Lerkvaleekul, Sirirat Charuvanij, Maynart Sukharomana, Kwanchai Pirojsakul, Malisa Kamolwatwong, Soamarat Vilaiyuk*

*** Correspondence:** Soamarat Vilaiyuk: soamarat21@hotmail.com

1 Supplementary Table

Table S1. Type of disease in 479 rheumatic patients in this study

Diseases	Number (%)
Juvenile idiopathic arthritis (JIA)	229 (47.81)
- Oligoarticular JIA	34 (14.85)
- Polyarticular JIA with RF negative	28 (12.23)
- Polyarticular JIA with RF positive	34 (14.85)
- Enthesitis-related arthritis	74 (32.31)
- Systemic JIA	54 (23.58)
- Psoriatic arthritis	1 (0.44)
- Undifferentiated arthritis	4 (1.75)
Connective tissue diseases	189 (39.46)
- Systemic lupus erythematosus	161 (85.19)
- Juvenile dermatomyositis	16 (8.47)
- Sjogren's syndrome	1 (0.53)
- Mixed connective tissue disease	1 (0.53)
- Overlapping syndromes	7 (3.70)
- Localized scleroderma	1 (0.53)
Vasculitis	42 (8.76)
- IgA vaculitis	27 (64.29)
- Takayasu arteritis	6 (14.29)
- BehÇet's disease	3 (7.14)
- Kawasaki disease	1 (2.38)
- Microscopic polyangitis	1 (2.38)
- Primary central nervous system vasculitis	1 (2.38)
- Cutaneous small vessel vasculitis	3 (7.14)
Other rheumatic diseases	19 (3.97)
- Uveitis	3 (15.79)
- Chronic recurrent multifocal osteomyelitis	2 (10.53)
- Catastrophic antiphospholipid syndrome	2 (10.53)
- Idiopathic pulmonary hemosiderosis	2 (10.53)
- Vogt–Koyanagi–Harada disease	3 (15.79)
- Nodular scleritis	1 (5.26)
- Deficiency of adenosine deaminase 2	1 (5.26)
- Inflammatory bowel disease	1 (5.26)
- Immune-mediated necrotizing myopathy	1 (5.26)
- Multiple sclerosis	1 (5.26)
- Neuromyelitis optica	1 (5.26)
- Sarcoidosis	1 (5.26)

RF, rheumatoid factor

Table S2. Side effects of the COVID-19 vaccination in each type of disease.

Side effect	JIA (n=205)	CTDs (n=176)	Vasculitis (n=32)	Others (n=16)
None, n (%)	157 (77.0)	137 (77.8)	25 (78.1)	13 (81.3)
Pain at injection site, n (%)	9 (4.4)	12 (6.8)	2 (6.3)	0 (0)
Fever, n (%)	28 (13.7)	20 (11.4)	4 (12.5)	2 (12.5)
Malaise, n (%)	6 (2.9)	3 (1.7)	1 (3.1)	1 (6.3)
Headache, n (%)	1 (0.5)	2 (1.1)	0 (0)	0 (0)
Rash, n (%)	1 (0.5)	0 (0)	0 (0)	0 (0)
Increased LFT, n (%)	0 (0)	1 (0.6)	0 (0)	0 (0)
Chest pain, n (%)	2 (1.0)	0 (0)	0 (0)	0 (0)
Diarrhea, n (%)	1 (0.5)	1 (0.6)	0 (0)	0 (0)

JIA, juvenile idiopathic arthritis; CTDs, connective tissue diseases

Table S3. Characteristics of COVID-19 infection between type of diseases (n = 245)

Characteristics	JIA (n=109)	CTDs (n=97)	Vasculitis (n=27)	Others (n=12)	*p*-value
Fever, n (%)	73 (67.0)	71 (73.2)	23 (85.2)	11 (91.7)	0.11
Cough, n (%)	61 (56.0)	63 (64.9)	18 (66.7)	10 (16.7)	0.21
Sore throat, n (%)	50 (45.9)	57 (58.8)	14 (51.9)	6 (50.0)	0.33
Nasal congestion, n (%)	55 (50.5)	53 (54.6)	15 (55.6)	7 (58.3)	0.90
Headache, n (%)	6 (5.5)	12 (12.4)	0 (0)	1 (8.3)	0.11
Pneumonia, n (%)	2 (1.8)	1 (1.0)	0 (0)	0 (0)	1.00
Diarrhea/vomiting, n (%)	4 (3.7)	6 (6.2)	1 (3.7)	1 (8.3)	0.64
Treatment of COVID-19 infection					
- Favipiravir, n (%)	44 (40.4)	55 (56.7)	6 (22.2)	7 (58.3)	0.005[a]
- Monopiravir, n (%)	3 (2.8)	3 (3.1)	2 (7.4)	0 (0)	0.63
- Herb (Andrographolide), n (%)	12 (11.0)	14 (14.4)	5 (18.5)	2 (16.7)	0.64
- Oxygen therapy, n (%)	2 (1.8)	1 (1.0)	0 (0)	0 (0)	1.00
- Admit ward, n (%)	3 (2.8)	10 (10.3)	0 (0)	0 (0)	0.07
- Home isolation, n (%)	106 (97.2)	87 (89.7)	27 (100)	12 (100)	
Outcome of COVID-19 infection					
- Recovery, n (%)	106 (97.2)	96 (99.0)	26 (96.3)	12 (100)	0.63
- Long COVID, n (%)	3 (2.8)	1 (1.0)	1 (3.7)	0 (0)	
Day of Recovery, n (%)	4.06 ± 2.56	4.91 ± 2.46	4.37 ± 2.50	3.83 ± 2.41	0.09

JIA, juvenile idiopathic arthritis; CTDs, connective tissue diseases.
[a] A value of $p < 0.05$ was considered to indicate statistical significance.

Table S4. Characteristics of COVID-19 infection between high-dose and low-dose prednisolone (n = 245)

Characteristics	High-dose prednisolone (n = 44)	Low-dose prednisolone (n = 201)	*p*-value
Fever, n (%)	27 (61.4)	151 (75.1)	0.09
Cough, n (%)	29 (65.9)	123 (61.2)	0.61
Sore throat, n (%)	20 (45.5)	107 (53.2)	0.41
Nasal congestion, n (%)	27 (61.4)	103 (51.2)	0.25
Headache, n (%)	3 (6.8)	16 (8.0)	1.00
Pneumonia, n (%)	0 (0)	3 (1.5)	1.00
Diarrhea/vomiting, n (%)	1 (2.3)	11 (5.5)	0.70
Treatment of COVID-19 infection			
- Favipiravir, n (%)	28 (63.6)	84 (41.8)	0.012[a]
- Monopiravir, n (%)	1 (2.3)	7 (3.5)	1.00
- Herb (Andrographolide), n (%)	3 (6.8)	30 (14.9)	0.22
- Oxygen therapy, n (%)	0 (0)	3 (1.5)	1.00
- Admit ward, n (%)	4 (9.1)	9 (4.5)	0.26
- Home isolation, n (%)	40 (90.0)	192 (95.5)	
Outcome of COVID-19 infection			
- Recovery, n (%)	44 (100)	196 (97.5)	0.66
- Long COVID, n (%)	0 (0)	5 (2.5)	
Day of Recovery, n (%)	4.50 ± 2.56	4.40 ± 2.52	0.82

[a] A value of $p < 0.05$ was considered to indicate statistical significance.

Table S5. Characteristics of COVID-19 infection between patients with and without biological treatment

Characteristics	With biological treatment (n = 32)	Without biological treatment (n = 213)	*p*-value
Fever, n (%)	20 (62.5)	158 (74.2)	0.20
Cough, n (%)	17 (53.1)	135 (63.4)	0.33
Sore throat, n (%)	16 (50.0)	111 (52.1)	0.85
Nasal congestion, n (%)	19 (59.4)	111 (52.1)	0.46
Headache, n (%)	0 (0)	19 (8.9)	0.15
Pneumonia, n (%)	0 (0)	3 (1.4)	1.00
Diarrhea/vomiting, n (%)	1 (3.1)	11 (5.2)	1.00
Treatment of COVID-19 infection			
- Favipiravir, n (%)	14 (43.8)	98 (46)	0.85
- Monopiravir, n (%)	2 (6.3)	6 (2.8)	0.28
- Herb (Andrographolide), n (%)	6 (18.8)	27 (12.7)	0.40
- Oxygen therapy, n (%)	0 (0)	3 (1.4)	1.00
- Admit ward, n (%)	0 (0)	13 (6.1)	0.23
- Home isolation, n (%)	32 (0.0)	200 (93.9)	
Outcome of COVID-19 infection			
- Recovery, n (%)	31 (96.9)	209 (98.12)	0.66
- Long COVID, n (%)	1 (3.1)	4 (1.9)	
Day of Recovery, n (%)	4.66 ± 2.72	4.38 ± 2.50	0.57

LFT, liver function test

Table S6. Characteristics of COVID-19 infection before and after receiving vaccination (n = 245)

Characteristics	Covid-19 infection before vaccination (n=41)	Covid-19 infection after vaccination (n=204)	*p*-value
Fever, n (%)	35 (85.4)	143 (70.1)	0.05
Cough, n (%)	23 (56.1)	129 (63.2)	0.48
Sore throat, n (%)	19 (46.3)	108 (53.0)	0.50
Nasal congestion, n (%)	23 (56.1)	107 (52.5)	0.73
Headache, n (%)	5 (12.2)	14 (6.9)	0.33
Pneumonia, n (%)	0 (0)	3 (1.5)	1.00
Diarrhea/vomiting, n (%)	2 (4.9)	10 (4.9)	1.00
Treatment of COVID-19 infection			
- Favipiravir, n (%)	17 (41.5)	95 (46.6)	0.61
- Monopiravir, n (%)	0 (0)	8 (3.9)	0.36
- Herb (Andrographolide), n (%)	6 (14.6)	27 (13.2)	1.00
- Oxygen therapy, n (%)	0 (0)	3 (1.5)	1.00
- Admit ward, n (%)	2 (4.9)	11 (5.4)	1.00
- Home isolation, n (%)	39 (95.1)	193 (94.6)	
Outcome of COVID-19 infection			
- Recovery, n (%)	41 (100)	199 (97.6)	0.66
- Long COVID, n (%)	0 (0)	5 (2.5)	
Day of Recovery, n (%)	4.51 ± 2.50	4.40 ± 2.53	0.80
